# Mitochondrial translation inhibition triggers ATF4 activation, leading to integrated stress response but not to mitochondrial unfolded protein response

**DOI:** 10.1042/BSR20201289

**Published:** 2020-11-24

**Authors:** Katsuhiko Sasaki, Takeshi Uchiumi, Takahiro Toshima, Mikako Yagi, Yura Do, Haruka Hirai, Ko Igami, Kazuhito Gotoh, Dongchon Kang

**Affiliations:** 1 Department of Clinical Chemistry and Laboratory Medicine, Graduate School of Medical Sciences, Kyushu University, 3-1-1, Maidashi, Higashi-ku, Fukuoka 812-8582, Japan; 2 Clinical Laboratory Department, Kyushu Pro-Search Limited Liability Partnership 4-1, Kyudaishimmachi, Nishi-ku, Fukuoka 819-0388, Japan; 3 Business Management Division, Clinical Laboratory Business Segment, LSI Medience Corporation, 13-4, Uchikanda 1-chome, Chiyoda-ku, Tokyo 101-8517, Japan; 4 Department of Health Sciences, Graduate School of Medical Sciences, Kyushu University, 3-1-1, Maidashi, Higashi-ku, Fukuoka 812-8582, Japan

**Keywords:** ATF4, integratad stress responce, mitochondria, mtUPR

## Abstract

Mitochondrial–nuclear communication, known as retrograde signaling, is important for regulating nuclear gene expression in response to mitochondrial dysfunction. Previously, we have found that p32/C1qbp-deficient mice, which have a mitochondrial translation defect, show endoplasmic reticulum (ER) stress response and integrated stress response (ISR) gene expression in the heart and brain. However, the mechanism by which mitochondrial translation inhibition elicits these responses is not clear. Among the transcription factors that respond to mitochondrial stress, activating transcription factor 4 (ATF4) is a key transcription factor in the ISR. Herein, chloramphenicol (CAP), which inhibits mitochondrial DNA (mtDNA)-encoded protein expression, induced eukaryotic initiation factor 2 α subunit (eIF2α) phosphorylation and ATF4 induction, leading to ISR gene expression. However, the expression of the mitochondrial unfolded protein response (mtUPR) genes, which has been shown in *Caenorhabditis elegans*, was not induced. Short hairpin RNA-based knockdown of ATF4 markedly inhibited the CAP-induced ISR gene expression. We also observed by ChIP analysis that induced ATF4 bound to the promoter region of several ISR genes, suggesting that mitochondrial translation inhibition induces ISR gene expression through ATF4 activation. In the present study, we showed that mitochondrial translation inhibition induced the ISR through ATF4 activation rather than the mtUPR.

## Introduction

Mitochondria are important not only for ATP production by oxidative phosphorylation (OXPHOS), but also for lipid metabolism, iron–sulfur cluster biogenesis, calcium signaling and apoptosis regulation [1]. Impaired mitochondrial translation causes severe combined respiratory chain dysfunctions, usually due to functional deficiency of all proteins encoded by the mitochondrial DNA (mtDNA). Thus, altered expression of mitochondrial proteins is increasingly associated with various human diseases [2].

Mitochondrial p32/C1qbp, which functions as an RNA and protein chaperone, interacts with mitochondrial mRNA and is indispensable for mitochondrial function through its regulation of mitochondrial translation in cultured mouse embryonic fibroblast (MEF) cell lines [3]. It has been reported that four individuals from unrelated families have biallelic mutations of C1QBP that cause a defect in mitochondrial energy metabolism [4]. Cardiomyocyte-specific deletion of p32 resulted in contractile dysfunction, cardiac dilatation and cardiac fibrosis, compared with hearts in control mice [5]. These mice showed decreased CoxI expression, decreased oxygen consumption rates and increased oxidative stress. We have found that the endoplasmic reticulum (ER) stress response and the expression levels of mitokines such as *Fgf21* and of integrated stress response (ISR) genes were significantly increased in these mice [5]. Neuron-specific deletion of p32 showed compromised mitochondrial respiration and shifted cellular metabolism toward glycolysis, leading to leukoencephalopathy and ER stress response [6]. However, the mechanism by which *p32* knockout (KO) elicits these responses in mice is not clear.

Along with the mitochondrial α-proteobacterial origin, the antibiotics which affect the ribosome inhibit mitochondrial translation [7]. Chloramphenicol (CAP) inhibits mitochondrial translation elongation by occupying the ribosomal A-site [8], but has quite a different effect on cell proliferation [7].

The ISR is an evolutionarily conserved homeostatic program activated by specific pathological states. These include iron deficiency, amino acid deprivation, viral infection and misfolded proteins within the ER. Though different, each of these stresses increases the phosphorylation of the transcription initiation factor, eukaryotic initiation factor 2 α subunit (eIF2α), and induces the activation of activating transcription factor 4 (ATF4) and the downstream gene expression, and protects against stress [9].

ATF4 plays a pivotal role in cell responses to stresses that induce eIF2α phosphorylation [10,11]. ATF4 target genes are involved in biosynthesis, folding and assembly of proteins, metabolism, transport of nutrients, protection against oxidative stress and regulation of apoptosis.

In response to accumulation of unfolded proteins or misfolded proteins at levels above the organelle’s chaperone capacity, the cell initiates a mitochondrial unfolded protein response (mtUPR) [12]. In *Caenorhabditis elegans*, this mtUPR is a mitochondrial signaling pathway leading to the induction of mitochondrial protective genes including mitochondrial molecular chaperones and proteases to re-establish protein homeostasis [13–15]. However, the mechanism of the mammalian mtUPR and regulated transcription factors is still unclear.

Interestingly, in human patients with mitochondrial myopathy caused by OXPHOS deficiencies, the cytokine, fibroblast growth factor 21 (FGF21), is secreted from muscle tissues, suggesting that OXPHOS dysfunction may mimic fasting and induce the secretion of the fasting-induced hormone, FGF21 [16]. These observations suggest that mtUPR, ISR and FGF21 expression act as protective responses against various mitochondria-related diseases.

In the present study, as ATF4 can be activated by mitochondrial translation inhibition, we analyzed the regulation of ATF4 expression such as mitochondrial translation inhibition, to better understand the signaling pathways triggered by mitochondrial dysfunction. The investigation of these stress signaling pathways may help to better characterize mitochondrial diseases associated with impaired mitochondrial translation.

## Materials and methods

### Cell preparation and retrovirus-mediated gene transfer

MEFs were maintained at 37°C and 5% CO_2_ in Dulbecco’s modified Eagle’s medium (DMEM; Sigma–Aldrich, St. Louis, MO, U.S.A.) supplemented with 10% FBS [3]. pSUPER retro puro GFP small hairpin RNA (shRNA) was a gift from Dr. John Gurdon from Cancer Research UK Gurdon Institute, Cambridge, UK (Addgene plasmid #30519). The retroviral vector, pSUPER retro puro, was used to generate a plasmid encoding GFP shRNA or ATF4 shRNA. The shRNA target sequences were: Mouse *ATF4* #1: 5′-AAGAGAAGGCAGATTCTCT-3′ and *ATF4* #2: 5′-GAGCATTCCTTTAGTTTAG-3′. These plasmids were transfected into Platinum-E packaging cells using FuGENE 6 transfection reagent (Roche Applied Science, Penzberg, Germany). The cell culture supernatants were harvested 48 h after transfection, supplemented with polybrene (5 μg/ml; Sigma–Aldrich) and used to infect MEFs.

### Reagents

CAP, doxycycline, and actinonin were obtained from Wako Pure Chemicals, Osaka, Japan. Antibodies against ATF4, p-eIF2α Ser^51^ (3795S), and eIF2α (9722S) were purchased from Cell Signaling Technology (Danvers, MA, U.S.A.). Anti-COXI (Ab14705) was from Abcam, Cambridge, U.K. COXIII and SDHA antibodies were from Thermo Fisher Scientific, Waltham, MA, U.S.A. The secondary antibodies, anti-mouse horseradish peroxidase (HRP)-conjugated IgG (#7076) and anti-rabbit HRP-conjugated IgG (#7074), were from Cell Signaling Technology.

### Real-time PCR analysis

Total RNA was extracted using the RNeasy kit (Qiagen, Hilden, Germany). cDNA was synthesized using total RNA, random hexamer primers and the PrimScript™ RT reagent Kit (TaKaRa, Otsu, Japan). The reverse transcription (RT) product was then subjected to real-time PCR analysis with SYBR Premix ExTaq™ II and the StepOnePlus Real-Time PCR System (Applied Biosystems, Carlsbad, CA, U.S.A.). Total mRNA from wild type and homozygous p32loxP/loxP mice (nestin-Cre^+/−^, p32loxP/loxP) were previously described [6]. Mouse experiments and protocols were performed in accordance with the guidelines of the animal ethics committee of Kyushu University Graduate School of Medicine, Japan (#A29-052-0). All experimental procedures confirmed to the Guide for the Care and Use of Laboratory Animals, eighth edition, updated by the U.S. National Research Council Committee in 2011 and approved by the guidelines by the Kyushu University Animal Care and Use Committee. The primer sequences used in the PCRs are listed in Supplementary Table S1. The expression level of each mRNA was normalized to the level of *18S* ribosomal RNA, obtained from the corresponding RT product.

### Immunoblotting analysis

For protein extraction, cells were kept on ice and scraped in lysis buffer (20 mM Tris, pH 7.4, 150 mM KCl, 1 mM EDTA and 1% Triton X-100) containing protease inhibitor cocktail (cOmplete™, Roche) and a phosphatase inhibitor mixture (1 mM NaVO_3_, 10 mM *p*-nitrophenyl phosphate, 10 mM β-glycerophosphate and 5 mM NaF). Clear lysates from MEF cells were retrieved from the supernatant of cell lysates after a 10-min centrifugation at 13000 rpm at 4°C. The protein concentration was determined using the Pierce 660 nm Protein Assay (Thermo scientific, 22660). Signals were visualized with HRP-conjugated anti-rabbit IgG and an enhanced chemiluminescence reagent (GE Healthcare, Piscataway, NJ, U.S.A.). Chemiluminescence was recorded and quantified with a chilled charge-coupled device camera (LAS 4000, GE Healthcare).

### Statistical analysis

Experiments were performed at least in triplicate, and the results shown are representative of *n*=3 independent biological experiments. Data are expressed as means ± SD or means ± SEM of the indicated number of replicates. Statistical analyses of the data were performed using two-tailed Student’s *t* test. *P*<0.05 was considered statistically significant (*P<0.05, **P<0.01 and ***P<0.001).

### Chromatin immunoprecipitation assay

Chromatin immunoprecipitation (ChIP) was performed using the MAGnify Chromatin Immunoprecipitation System (49–2024; Invitrogen, Carlsbad, CA, U.S.A.) according to the manufacturer’s instructions with some modifications [17]. In brief, CAP-treated MEFs were cross-linked with 1% (w/v) formaldehyde for 10 min and lysed in the buffer provided. Nuclear extracts from 3 × 10^6^ were used per immunoprecipitation reaction. Sonicated nuclear extracts were immunoprecipitated for 2 h at 4°C with anti-ATF4 (sc200×, 3 μg) or IgG isotype negative control (sc2027, 3 μg) antibodies (Cell Signaling Technology). DNA was eluted and purified as previously described [18]. Eluted DNA was quantified with quantitative PCR (qPCR) using SYBR Green chemistry (StepOnePlus; Applied Biosystems, Foster City, CA, U.S.A.). The primer sequences used in the PCRs are listed in Supplementary Table S2.

### Pulse-labeling of mitochondrial translation products

Mitochondrial translation products were pulse-labeled *in vitro* with [^35^S]-(methionine and cysteine) (GE Healthcare). In experiments where the label was chased, MEF cells were incubated for 6 min with 100 μg/ml emetine or 250 μg/ml CAP prior to labeling for 60 min. Labeled cells were then rinsed with an isotonic buffer (25 mM Tris-HCl, pH 7.4, 137 mM NaCl, 10 mM KCl and 0.7 mM Na_2_HPO_4_). After centrifugation at 1150×***g*** for 5 min, cell pellets were resuspended in loading buffer consisting of 93 mM Tris-HCl, pH 6.7, 7.5% glycerol, 1% SDS, 0.25 mg/ml Bromophenol Blue and 3% mercaptoethanol. The total lysate was then subjected to 15% SDS/PAGE for 3 h at 180 V. Gels were examined using a Fujifilm BAS-2500 Bio Imaging Analyzer (Fujifilm, Tokyo, Japan) [3].

### Mating of transgenic mice

Mouse experiments and protocols were performed in accordance with the guidelines of the animal ethics committee of Kyushu University Graduate School of Medicine, Japan (#A29-052-0). All experimental procedures confirmed to the Guide for the Care and Use of Laboratory Animals, eighth edition, updated by the U.S. National Research Council Committee in 2011 and approved by the guidelines by the Kyushu University Animal Care and Use Committee. p32cKO mice (nestin-Cre^+/−^, p32loxP/loxP) were generated as previously reported [6].

## Results

### CAP inhibits mitochondrial translation

**Figure 1:**
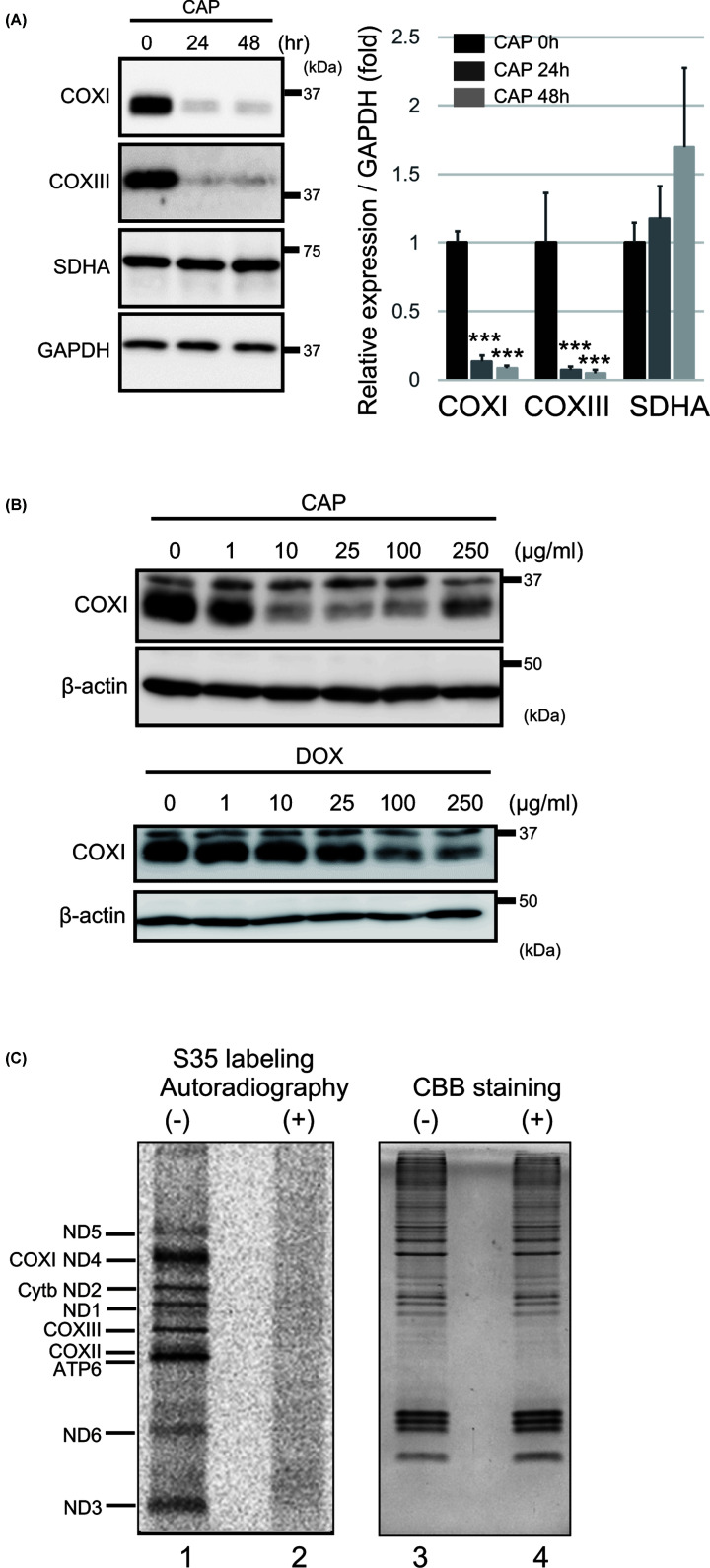
CAP inhibits mitochondrial translation (**A**) CAP-mediated inhibition of mitochondrial translation efficiency was monitored by Western blot analysis of the abundance of CoxI and CoxIII proteins encoded by mtDNA. MEFs were incubated with control or 100 μg/ml CAP-containing medium for 24 and 48 h. Immunodetection of GAPDH and SDHA was used as the loading control. All experiments were done in triplicate. Quantification is shown in the right panel. ***P<0.005. (**B**) Antibiotics such as Chloramphenicol (CAP)- and Doxycycline (DOX)-mediated CoxI expression was monitored by Western blot analysis for 48 h in MEF cells. Immunodetection of β-actin was used as the loading control. (**C**) *In vivo* mitochondrial translation was performed for 60 min. The products were labeled during the reaction with a mixture of [^35^S]-methionine and [^35^S]-cysteine in the presence of emetine and/or CAP, and then detected by autoradiography after SDS/PAGE (left panel). Deficient translation was observed in CAP-treated MEFs. Equal loading was confirmed by CBB staining (right panel). The proteins indicated are representative proteins based on their molecular weight.

First, we investigated whether CAP directly affects mitochondrial translation of MEF cells. We observed that CAP treatment decreased the expression of CoxI and CoxIII, which are encoded by mtDNA, in a time- and dose-dependent manner (Figure 1A,B). Another antibiotic, doxycycline, also decreased the expression of the mitochondrial-encoded core subunits of CoxI (Figure 1B). However, the GAPDH, β-actin and SDHA expression did not decrease. Next, to investigate the defect in mitochondrial protein synthesis, MEFs were pulse-labeled with a mixture of [^35^S]-methionine and [^35^S]-cysteine. Electrophoresis of whole-protein extracts from emetine-treated cells, which is a specific inhibitor of cytoplasmic protein synthesis, showed radioactive bands in the control (Figure 1C, lane 1). This profile clearly showed at least nine products of mitochondrial protein synthesis. However, the synthesis of these proteins was completely inhibited by CAP pretreatment for 12 h (Figure 1C, lane 2), confirming that CAP inhibits mitochondrial protein synthesis.

### CAP induces the ER stress response

**Figure 2:**
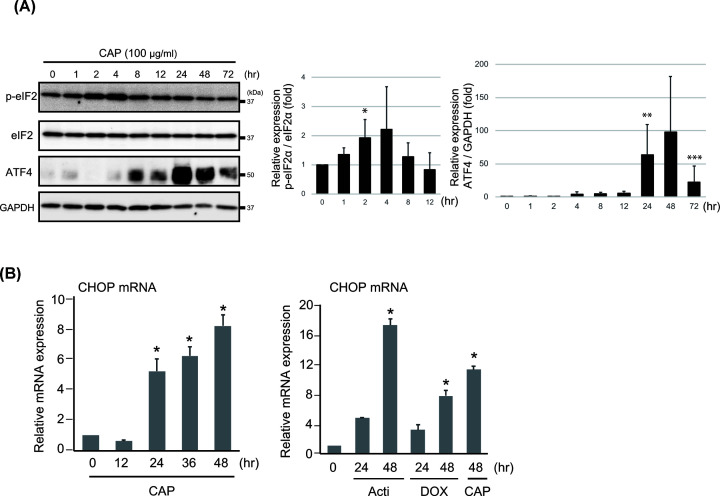
CAP induces the ER stress response (**A**) The levels of eIF2α phosphorylation at Ser^51^ and ATF4 expression were evaluated in MEFs incubated with or without 100 μg/ml CAP for 48 h. The abundance of both phosphorylated and total eIF2α was assessed by Western blotting. Immunodetection of β-actin was used as the loading control. All experiments were done in triplicate. Quantification is shown in the right panel. *P<0.05 **P<0.01, ***P<0.001 vs control. (**B**) *Chop-10* mRNA abundance was evaluated by RT-qPCR in MEFs treated with CAP. *Chop-10* mRNA abundance was also evaluated in MEFs treated with the antibiotics, actinonin (50 μM), doxycycline (100 μg/ml) and CAP (100 μg/ml), for 48 h. *Chop-10* mRNA was normalized to *18S* rRNA, which was the loading control. All experiments were done in triplicate; *P<0.05.

Previously, we observed that knockout of the mitochondrial protein, p32/C1qbp, in mice caused the dysfunction of mitochondrial translation and ER stress induction, leading to induction of ISR genes [5,6]. As shown in Figure 2, eIF2α phosphorylation at Ser^51^, a well-established indicator of ER stress, was increased 2–4 h after CAP treatment MEF cells. Furthermore, ATF4 was up-regulated in MEFs from 4 to 48 h in response to CAP (Figure 2A), similar to *p32*^−/−^ MEFs, suggesting that mitochondrial translation inhibition induced eIF2α phosphorylation and ATF4 expression.

Several studies have described the induction of CHOP-10 upon different kinds of mitochondrial dysfunction [19]. Thus, we next assessed *CHOP-10* mRNA expression. The mRNA abundance of *CHOP-10* assessed by RT-qPCR indicated that it was significantly increased after CAP treatment in a time-dependent manner (Figure 2B). We also observed that other antibiotics, such as actinonin, peptide deformylase (PDF) inhibitor and doxycycline, induced *CHOP-10* expression (Figure 2B).

### Mitochondrial dysfunction triggers ISR gene expression

**Figure 3:**
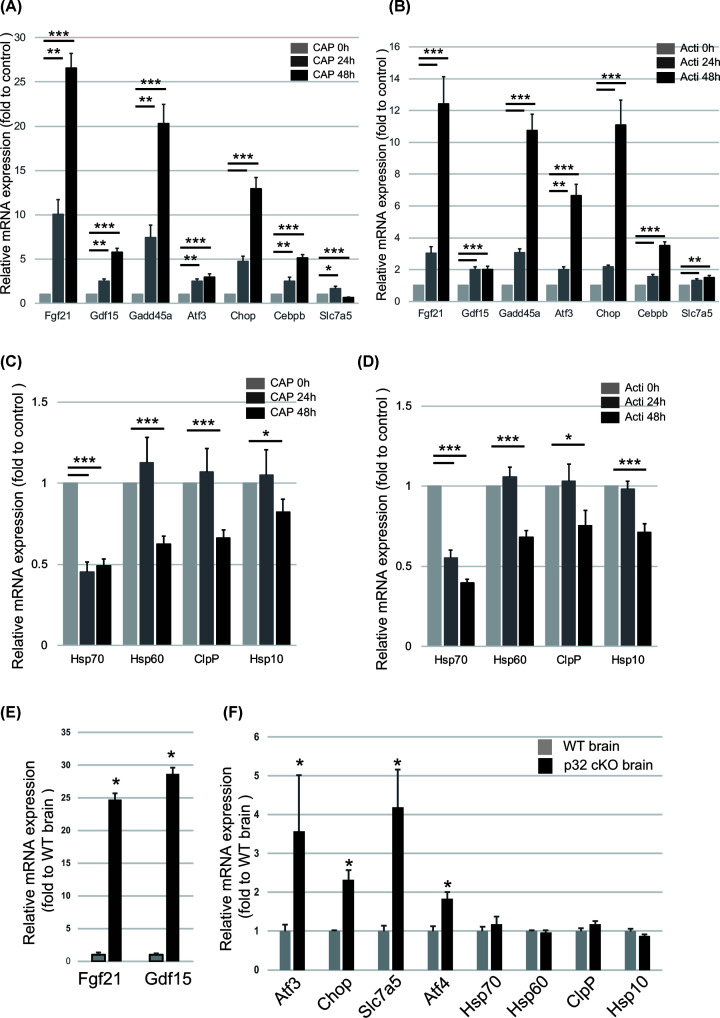
Interfering with mitochondrial translation in wild type MEFs triggers the ISR, but not the mtUPR (**A,B**) The mRNA abundance of the ISR-related gene markers, *Trib3, Atf3* and *Gadd45*, in MEFs treated with 50 μM CAP (A) or 50 μM Acti (actinonin) (B) for 48 h was assessed by RT-qPCR. Results are expressed the mean ± SD of three independent experiments. (**C,D**) The abundance of *ClpP, Hsp70, Hsp10* and *Hsp60* mRNA in MEFs treated with CAP (C) or Actinonin (D) for 24 and 48 h was assessed by RT-qPCR. Results are expressed as the mean ± SD of three independent experiments. (**E**) FGF21 and GDF15 gene expression were up-regulated in 6-week-old p32cKO brain compared with control brain. Results are expressed as the mean ± SD of three independent experiments. (**F**) qRT-PCR analysis confirmed that ISR gene is up-regulated, but not mtUPR marker gene expression in 6-week-old p32cKO brain compared with control brain. Results are expressed the mean ± SD of three independent experiments. *P<0.05 **P<0.01, ***P<0.005 vs. control.

ATF4 is induced by eIF2α phosphorylation and regulates the ISR to protect cells from metabolic consequences of ER stress. mtUPR has not been described in mammals, whereas cell signaling activated by accumulation of unfolded proteins has been described in *C. elegans* [13]. To investigate whether CAP induces ISR gene expression or mtUPR, we performed RT-qPCR analysis in MEF cells. We observed ISR activation (e.g., *Ddit3/Chop-10, Fgf21* and *GDF15*) after CAP treatment (Figure 3A). Actinonin also induced the ER stress response as shown by *ATF3* and *CHOP-10* expression, suggesting that mitochondrial translation inhibition is involved in ISR (Figure 3B). However, the mRNA expression of the mtUPR markers, *Hsp70, Hsp60, ClpP* and *Hsp10*, was not significantly up-regulated in CAP or actinonin-treated MEFs (Figure 3C,D). Next we evaluated the gene expression in p32 knockout mouse brain that inhibits mitochondrial translation. We also observed that increased levels of ISR gene mRNA, but not mtUPR marker gene expression in p32 conditional knockout mouse brain (Figure 3E,F). These results indicated that mtUPR was not induced in mammalian cells after mitochondrial translation inhibition.

### *ATF4* knockdown suppresses gene expression

**Figure 4:**
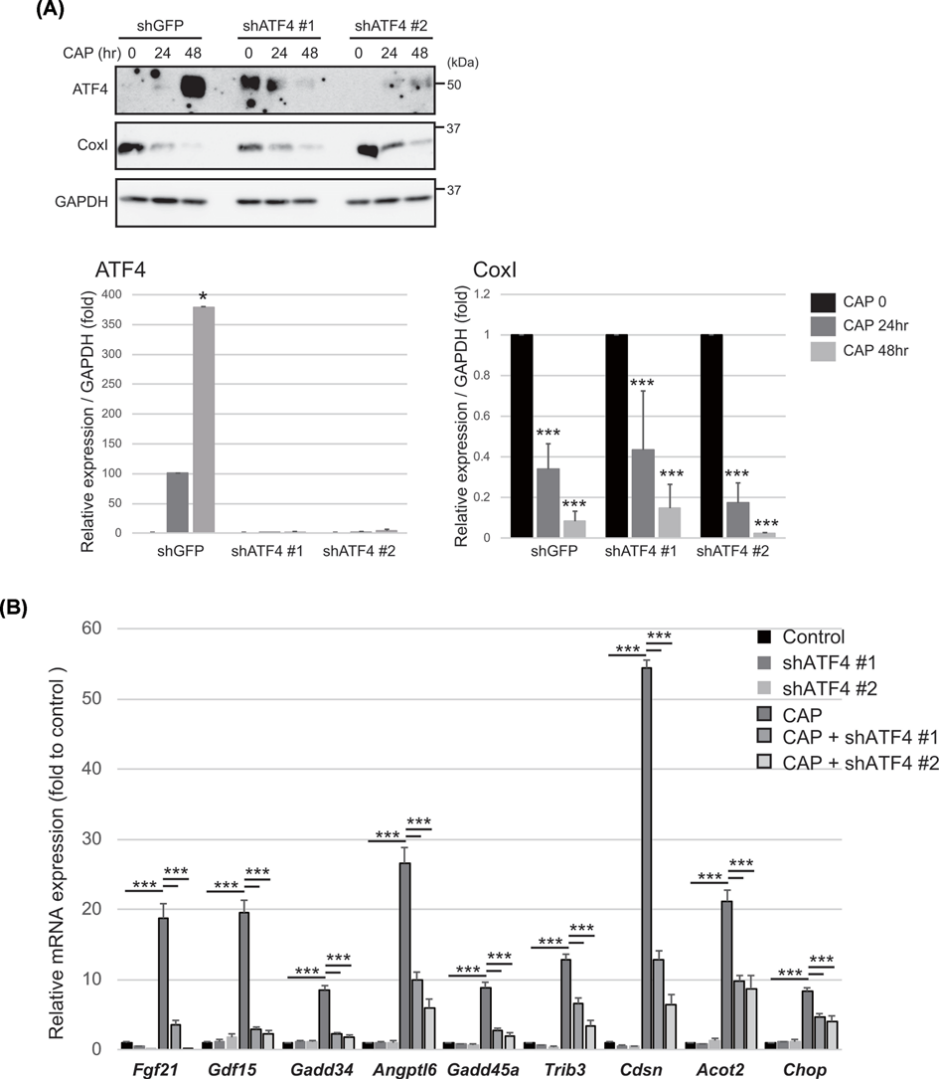
Effect of ATF4 silencing on CAP-induced ISR gene expression (**A**) MEFs stably transfected with either non-targeting shRNA (shGFP) or with two different shRNAs against *ATF4* (shATF4) for 24 h was replaced with control medium or with medium containing CAP (100 μg/ml) for 24 h. CAP induced CoxI decline were assessed by Western blotting. Immunodetection of GAPDH was used as the loading control. All experiments were done in triplicate. Quantification is shown in the right panel.*P<0.05, ***P<0.005 vs control.** (B**) MEFs which were stably transfected were incubated with or without CAP (100 μg/ml) for 12 h. The mRNA expression of ISR and mtUPR-related genes was evaluated by RT-qPCR. Results are expressed as fold change to control and are expressed the mean ± SD of three independent experiments. ***P<0.005 vs. control.

To examine the direct effect of ATF4 pathway activation on ISR gene expression in CAP-treated MEFs, we developed two cell lines using hairpin RNA-based knockdown of ATF4 (shATF4#1 and shATF4#2). Because ATF4 is not expressed in steady state, we confirmed the reduced ATF4 expression after treatment with CAP. We observed the ATF4 is induced by CAP treatment, but not in ATF4 knockdown MEF cells (Figure 4A).

Next, we examined the expression of ISR genes (e.g., *Fgf21, Gdf15, Gadd34, Angptl6, Gadd45a, Trib3, Cdsn, Acot2* and *Chop-10*) after CAP treatment. *ATF4* knockdown cells had decreased ISR gene expression in the presence of CAP in MEF cells (Figure 4B). These results indicate that inhibition of mitochondrial translation also regulates CAP-mediated ISR gene expression through ATF4 activation, similar to the loss of p32.

To study the physiological function of the ISR induced during mitochondrial translation deficiency, we performed the CoxI protein expression in control and shATF4 cells after CAP treatment. CoxI expression were similarly reduced in shGFP and shATF4 cells (Figure 4A), suggesting that ATF4-mediated ISR signaling did not affect the mitochondrial translation and may affect intracellular metabolic process.

### ATF4 binds to ISR gene promoters upon CAP treatment

**Figure 5:**
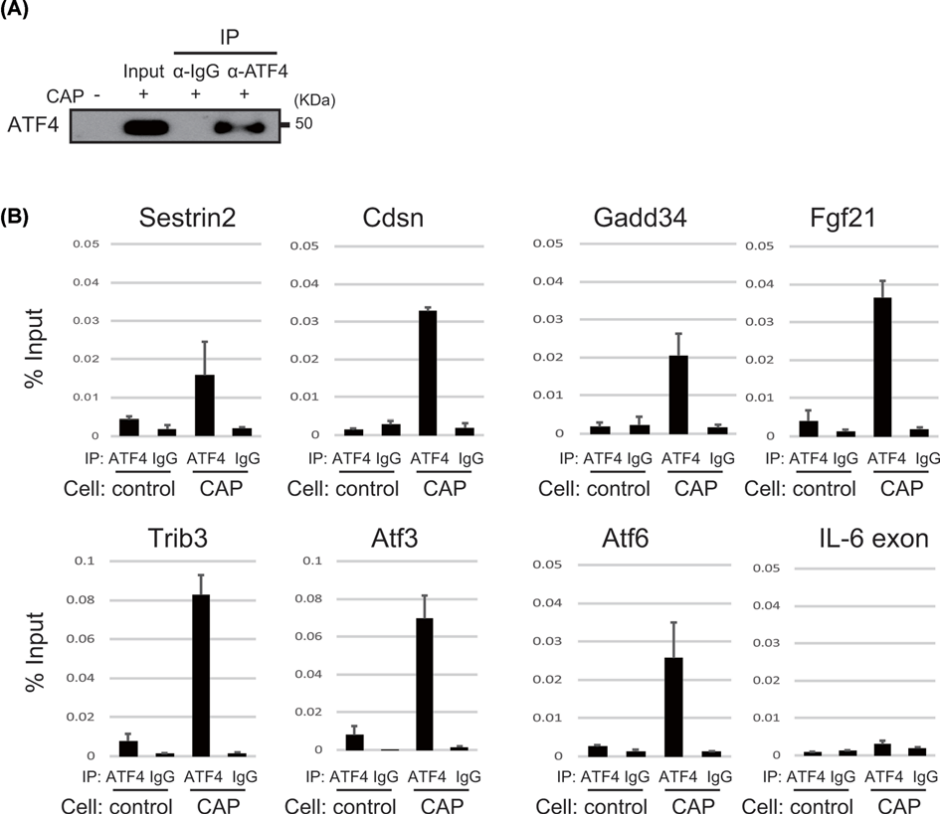
ATF4 directly activates ISR gene promoters (**A**) ATF4 was immunoprecipitated with anti-ATF4 antibodies after formaldehyde cross-linking of MEFs treated with CAP. We used anti IgG antibody for negative control. (**B**) Chromatin was immunoprecipitated, followed by quantitation by PCR analysis of the region containing the CARE site in ISR gene promoters (*Fgf21, Gadd45, Sestrin2 Cdsn, Trib3, Atf3, Atf6*). *Il-6* exon 2 was used as a negative control. Results are expressed as the mean ± SD of three independent experiments.

Characterization of individual genes as well as ChIP analysis revealed a consensus ATF4 binding domain: C/EBP-ATF responsive element (CARE) sequence (5′-TGATGXAAX-3′) [20]. To confirm the direct recruitment of ATF4 to the promoters of ISR genes such as *Trib3*, we performed a ChIP assay using anti-ATF4 antibodies and CAP-treated MEFs. Non-treated MEFs and anti-IgG antibodies were used as a negative control. We observed that ATF4 was immunoprecipitated with the ATF4 antibody only in CAP-treated MEFs (Figure 5A). Furthermore, signals from the promoter region of genes such as *Sestrin2, Cdsn, Gadd34, Trib3, Atf6* and *Atf3*, containing CARE, were significantly increased upon treatment with CAP (Figure 5B). We did not observe binding of ATF4 to the *IL-6* exon region, which does not have an ATF4-binding site (Figure 5B). These data suggest that ATF4 directly activates the promoter of ISR genes such as *Fgf21, Sestrin2, Cdsn, Gadd34, Trib3, Atf6* and *Atf3*.

## Discussion

Coordinated expression of both genomes and communication between the mitochondrion and the nucleus are critical for maintaining proper cell function and protecting the cell from oxidative stress. Our study showed that mitochondrial translation inhibition led to ER stress and ATF4-dependent ISR gene expression. The major new findings of the present study are: (1) mitochondrial translation inhibition induced elF2α phosphorylation and ATF4 induction. (2) Induced ATF4 directly bound to the promoter of ISR genes and ATF4 silencing did not induce CAP-dependent ISR gene induction. (3) In mammalian cells, mitochondrial translation inhibition did not induce mtUPR, which has been observed in *C. elegans*.

It has been reported that transcription regulators are key players in mitochondrion-to-nucleus communication, as they orchestrate the expression of nuclear genes in response to both external challenges and organelle dysfunction, to regulate processes such as mitochondrial biogenesis, cell proliferation, metabolism and apoptosis [21]. In our previous study, decreased heart function *in vivo* was observed in *p32*cKO mice based on both echocardiography and gene expression of heart failure markers. However, these mice did not die until the age of approximately 1 year [5]. ATF4 is a master regulator of the ISR. In ISR, a variety of stresses, including amino acid starvation, glucose starvation, ER stress, hypoxia and oxidative stress, can promote eIF2α phosphorylation, followed by ATF4 up-regulation, which activates the expression of stress-responsive genes [10,22]. These results suggest that the ATF4-induced ISR gene expression was protective against heart failure for approximately 1 year.

In *C. elegans*, CHOP-10 is associated with the UPR of the ER (erUPR) [23]. However, Hoogenraad and colleagues have reported that CHOP-10 is also activated in mtUPR [12]. Although several mechanisms of mtUPR have been recently described in *C. elegans* [13], the mammalian counterpart is less defined. In addition, among the different gene markers that are potentially overexpressed in response to mitochondrial translation inhibition in mammals [24], it has been reported that only the genes encoding ClpP and HSPD1 have been consistently assessed in subsequent studies dealing with mito–nuclear protein imbalance [25,26]. Considering our results, it appears that canonical mtUPR after mitochondrial translation inhibition in *C. elegans* is different from that in mammalian cells, suggesting that in mammals there is another response pathway against mitochondrial dysfunction.

FGF21 is generally considered to be a hepatic hormone involved in the control of glucose, lipid and energy metabolism. Recently, Gdf15 has been shown to be a novel diagnostic marker for mitochondrial diseases [27,28]. FGF21 released by cardiomyocytes acts as an antioxidant factor in the heart, preventing accumulation of reactive oxygen species induced by inflammatory or hypertrophic conditions [29]. The FGF21 functions promote the maintenance of health, and thus increase the lifespan [30,31]. Taken together, our findings suggest that the ISR may also contribute to the induction of Fgf21 and Gdf15 expression in response to defective energy metabolism. Additionally, the ISR may play a role in protecting the heart in association with alterations in the hypertrophic response [32,33], and therefore it may extend the lifespan. It has been reported that neuron-specific, inducible *in vivo* ablation of the mitochondrial fission protein, Drp1, causes ER stress, leading to activation of the ISR, culminating in neuronal expression of the cytokine, Fgf21 [34]. We also found that neural-specific p32 depletion induced ATF4-dependent Fgf21 expression. However, *p32*-KO mice died young, suggesting a different mechanism is involved.

In normal cells, ATF3 and ATF6 are highly regulated transcription factors that are implicated in a wide range of pathophysiological conditions. Both proteins are induced by several stress-induced signaling pathways, including ER stress and amino acid deficiency, and are regulated by an ATF4-dependent pathway [20]. ATF4 is a major hub of the cellular adaptive–response network including protein chaperons, cell survival and metabolic homeostasis. Sestrins have antioxidant functions and enforce homeostasis, stress resistance and protection against deleterious oxidation-induced DNA modifications and mutations [35]. We found that up-regulation of SESN2 by mitochondrial dysfunction provides homeostatic feedback that attenuates the biosynthetic process during transient loss of energy supply from the mitochondria. Hence, we suggest that these responses are important for adaptive and viable responses of the cell.

Using a multiomics approach in mammalian cells treated with four types of mitochondrial stressors (CCCP, DOX, Actinonin and MitBlock6), they also found that ATF4 as the main regulator of ISR, but not UPR [36]. Inhibiting mitochondrial translation activated an ATF4-dependent cascade leading to coordinated repression of cytosolic translation and activate ISR gene expression, which could be targeted to promote longevity [37]. In eukaryotic cells, the ER and the mitochondria have a tight interplay, which is structurally and functionally modulated through a tether formation at specific subdomains of the ER membrane, described as mitochondria-associated membranes (MAMs) [38]. Mitochondrial translation inhibition by antibiotics, which induces a mito–nuclear imbalance and mitochondrial dysfunction [26], can also trigger ATF4–mediated expression of stress genes [39]. However, the mechanisms linking MAM integrity and ER stress after mitochondrial translation dysfunction remain poorly understood.

In the present study, we discovered that the mitochondrial translation inhibition immediately induced elF2α phosphorylation and ATF4 induction, and activated ATF4 directly induced ISR gene expression. ATF4 recruitment to the CARE site within the promoter of various ISR genes was responsible for transcriptional control of ISR genes.

## Supplementary Material

Supplementary Tables S1-S2

## Data Availability

All the data are present in the manuscript. All the data are available from the corresponding author Takeshi Uchiumi (uchiumi@cclm.med.kyushu-u.ac.jp) under reasonable request.

## Abbreviations

ATF4: activating transcription factor 4
CAP: chloramphenicol
CARE: C/EBP-ATF responsive element
eIF2α: eukaryotic initiation factor 2 α subunit
ER: endoplasmic reticulum
FGF21: fibroblast growth factor 21
HRP: horseradish peroxidase
ISR: integrated stress response
KO: knockout
MAM: mitochondria-associated membrane
MEF: mouse embryonic fibroblast
mtDNA: mitochondrial DNA
mtUPR: mitochondrial unfolded protein response
OXPHOS: oxidative phosphorylation
qPCR: quantitative PCR
RT: reverse transcription
shRNA: small hairpin RNA

## References

[1] Wallace D.C. (2005) A mitochondrial paradigm of metabolic and degenerative diseases, aging, and cancer: a dawn for evolutionary medicine. Annu. Rev. Genet. 39, 359–407 10.1146/annurev.genet.39.110304.095751 16285865 PMC2821041

[2] Boczonadi V. and Horvath R. (2014) Mitochondria: impaired mitochondrial translation in human disease. Int. J. Biochem. Cell Biol. 48, 77–84 10.1016/j.biocel.2013.12.011 24412566 PMC3988845

[3] Yagi M., Uchiumi T., Takazaki S., Okuno B., Nomura M., Yoshida S. et al. (2012) p32/gC1qR is indispensable for fetal development and mitochondrial translation: importance of its RNA-binding ability. Nucleic Acids Res. 40, 9717–9737 10.1093/nar/gks774 22904065 PMC3479211

[4] Feichtinger R.G., Olahova M., Kishita Y., Garone C., Kremer L.S., Yagi M. et al. (2017) Biallelic C1QBP mutations cause severe neonatal-, childhood-, or later-onset cardiomyopathy associated with combined respiratory-chain deficiencies. Am. J. Hum. Genet. 101, 525–538 10.1016/j.ajhg.2017.08.015 28942965 PMC5630164

[5] Saito T., Uchiumi T., Yagi M., Amamoto R., Setoyama D., Matsushima Y. et al. (2017) Cardiomyocyte-specific loss of mitochondrial p32/C1qbp causes cardiomyopathy and activates stress responses. Cardiovasc. Res. 113, 1173–1185 10.1093/cvr/cvx095 28498888

[6] Yagi M., Uchiumi T., Sagata N., Setoyama D., Amamoto R., Matsushima Y. et al. (2017) Neural-specific deletion of mitochondrial p32/C1qbp leads to leukoencephalopathy due to undifferentiated oligodendrocyte and axon degeneration. Sci. Rep. 7, 15131 10.1038/s41598-017-15414-5 29123152 PMC5680297

[7] van den Bogert C., van Kernebeek G., de Leij L. and Kroon A.M. (1986) Inhibition of mitochondrial protein synthesis leads to proliferation arrest in the G1-phase of the cell cycle. Cancer Lett. 32, 41–51 10.1016/0304-3835(86)90037-6 3017546

[8] Bulkley D., Innis C.A., Blaha G. and Steitz T.A. (2010) Revisiting the structures of several antibiotics bound to the bacterial ribosome. Proc. Natl. Acad. Sci. U.S.A. 107, 17158–17163 10.1073/pnas.1008685107 20876130 PMC2951403

[9] Dalton L.E., Healey E., Irving J. and Marciniak S.J. (2012) Phosphoproteins in stress-induced disease. Prog. Mol. Biol. Transl. Sci. 106, 189–221 10.1016/B978-0-12-396456-4.00003-1 22340719

[10] Teske B.F., Wek S.A., Bunpo P., Cundiff J.K., McClintick J.N., Anthony T.G. et al. (2011) The eIF2 kinase PERK and the integrated stress response facilitate activation of ATF6 during endoplasmic reticulum stress. Mol. Biol. Cell 22, 4390–4405 10.1091/mbc.e11-06-0510 21917591 PMC3216664

[11] Wengrod J.C. and Gardner L.B. (2015) Cellular adaptation to nutrient deprivation: crosstalk between the mTORC1 and eIF2alpha signaling pathways and implications for autophagy. Cell Cycle 14, 2571–2577 10.1080/15384101.2015.1056947 26039820 PMC4614032

[12] Zhao Q., Wang J., Levichkin I.V., Stasinopoulos S., Ryan M.T. and Hoogenraad N.J. (2002) A mitochondrial specific stress response in mammalian cells. EMBO J. 21, 4411–4419 10.1093/emboj/cdf445 12198143 PMC126185

[13] Pellegrino M.W., Nargund A.M. and Haynes C.M. (2013) Signaling the mitochondrial unfolded protein response. Biochim. Biophys. Acta 1833, 410–416 10.1016/j.bbamcr.2012.02.019 22445420 PMC3393825

[14] Haynes C.M., Petrova K., Benedetti C., Yang Y. and Ron D. (2007) ClpP mediates activation of a mitochondrial unfolded protein response in C. elegans. Dev. Cell 13, 467–480 10.1016/j.devcel.2007.07.016 17925224

[15] Haynes C.M., Yang Y., Blais S.P., Neubert T.A. and Ron D. (2010) The matrix peptide exporter HAF-1 signals a mitochondrial UPR by activating the transcription factor ZC376.7 in C. elegans. Mol. Cell 37, 529–540 10.1016/j.molcel.2010.01.015 20188671 PMC2846537

[16] Suomalainen A., Elo J.M., Pietilainen K.H., Hakonen A.H., Sevastianova K., Korpela M. et al. (2011) FGF-21 as a biomarker for muscle-manifesting mitochondrial respiratory chain deficiencies: a diagnostic study. Lancet Neurol. 10, 806–818 10.1016/S1474-4422(11)70155-7 21820356 PMC7568343

[17] Dahl J.A. and Collas P. (2008) A rapid micro chromatin immunoprecipitation assay (microChIP). Nat. Protoc. 3, 1032–1045 10.1038/nprot.2008.68 18536650

[18] Uchiumi T., Ohgaki K., Yagi M., Aoki Y., Sakai A., Matsumoto S. et al. (2010) ERAL1 is associated with mitochondrial ribosome and elimination of ERAL1 leads to mitochondrial dysfunction and growth retardation. Nucleic Acids Res. 38, 5554–5568 10.1093/nar/gkq305 20430825 PMC2938226

[19] Moisoi N., Klupsch K., Fedele V., East P., Sharma S., Renton A. et al. (2009) Mitochondrial dysfunction triggered by loss of HtrA2 results in the activation of a brain-specific transcriptional stress response. Cell Death Differ. 16, 449–464 10.1038/cdd.2008.166 19023330

[20] Han J., Back S.H., Hur J., Lin Y.H., Gildersleeve R., Shan J. et al. (2013) ER-stress-induced transcriptional regulation increases protein synthesis leading to cell death. Nat. Cell Biol. 15, 481–490 10.1038/ncb2738 23624402 PMC3692270

[21] Ryan M.T. and Hoogenraad N.J. (2007) Mitochondrial-nuclear communications. Annu. Rev. Biochem. 76, 701–722 10.1146/annurev.biochem.76.052305.091720 17227225

[22] Harding H.P., Zhang Y., Zeng H., Novoa I., Lu P.D., Calfon M. et al. (2003) An integrated stress response regulates amino acid metabolism and resistance to oxidative stress. Mol. Cell 11, 619–633 10.1016/S1097-2765(03)00105-9 12667446

[23] Schroder M. and Kaufman R.J. (2005) The mammalian unfolded protein response. Annu. Rev. Biochem. 74, 739–789 10.1146/annurev.biochem.73.011303.074134 15952902

[24] Aldridge J.E., Horibe T. and Hoogenraad N.J. (2007) Discovery of genes activated by the mitochondrial unfolded protein response (mtUPR) and cognate promoter elements. PLoS ONE 2, e874 10.1371/journal.pone.0000874 17849004 PMC1964532

[25] Houtkooper R.H., Mouchiroud L., Ryu D., Moullan N., Katsyuba E., Knott G. et al. (2013) Mitonuclear protein imbalance as a conserved longevity mechanism. Nature 497, 451–457 10.1038/nature12188 23698443 PMC3663447

[26] Mouchiroud L., Houtkooper R.H., Moullan N., Katsyuba E., Ryu D., Canto C. et al. (2013) The NAD(+)/Sirtuin pathway modulates longevity through activation of mitochondrial UPR and FOXO signaling. Cell 154, 430–441 10.1016/j.cell.2013.06.016 23870130 PMC3753670

[27] Fujita Y., Ito M., Kojima T., Yatsuga S., Koga Y. and Tanaka M. (2015) GDF15 is a novel biomarker to evaluate efficacy of pyruvate therapy for mitochondrial diseases. Mitochondrion 20, 34–42 10.1016/j.mito.2014.10.006 25446397

[28] Yatsuga S., Fujita Y., Ishii A., Fukumoto Y., Arahata H., Kakuma T. et al. (2015) Growth differentiation factor 15 as a useful biomarker for mitochondrial disorders. Ann. Neurol. 78, 814–823 10.1002/ana.24506 26463265 PMC5057301

[29] Di Lisa F. and Itoh N. (2015) Cardiac Fgf21 synthesis and release: an autocrine loop for boosting up antioxidant defenses in failing hearts. Cardiovasc. Res. 106, 1–3 10.1093/cvr/cvv050 25712960

[30] Inagaki T., Lin V.Y., Goetz R., Mohammadi M., Mangelsdorf D.J. and Kliewer S.A. (2008) Inhibition of growth hormone signaling by the fasting-induced hormone FGF21. Cell Metab. 8, 77–83 10.1016/j.cmet.2008.05.006 18585098 PMC2575072

[31] Zhang Y., Xie Y., Berglund E.D., Coate K.C., He T.T., Katafuchi T. et al. (2012) The starvation hormone, fibroblast growth factor-21, extends lifespan in mice. Elife 1, e00065 10.7554/eLife.00065 23066506 PMC3466591

[32] Baird T.D. and Wek R.C. (2012) Eukaryotic initiation factor 2 phosphorylation and translational control in metabolism. Adv. Nutr. 3, 307–321 10.3945/an.112.002113 22585904 PMC3649462

[33] Pakos-Zebrucka K., Koryga I., Mnich K., Ljujic M., Samali A. and Gorman A.M. (2016) The integrated stress response. EMBO Rep. 17, 1374–1395 10.15252/embr.201642195 27629041 PMC5048378

[34] Restelli L.M., Oettinghaus B., Halliday M., Agca C., Licci M., Sironi L. et al. (2018) Neuronal mitochondrial dysfunction activates the integrated stress response to induce fibroblast growth factor 21. Cell Rep. 24, 1407–1414 10.1016/j.celrep.2018.07.023 30089252 PMC6092266

[35] Sablina A.A., Budanov A.V., Ilyinskaya G.V., Agapova L.S., Kravchenko J.E. and Chumakov P.M. (2005) The antioxidant function of the p53 tumor suppressor. Nat. Med. 11, 1306–1313 10.1038/nm1320 16286925 PMC2637821

[36] Quiros P.M., Prado M.A., Zamboni N., D’Amico D., Williams R.W., Finley D. et al. (2017) Multi-omics analysis identifies ATF4 as a key regulator of the mitochondrial stress response in mammals. J. Cell Biol. 216, 2027–2045 10.1083/jcb.201702058 28566324 PMC5496626

[37] Molenaars M., Janssens G.E., Williams E.G., Jongejan A., Lan J., Rabot S. et al. (2020) A conserved mito-cytosolic translational balance links two longevity pathways. Cell Metab. 31, 549.e7–563.e7 10.1016/j.cmet.2020.01.011 32084377 PMC7214782

[38] Marchi S., Giorgi C., Oparka M., Duszynski J., Wieckowski M.R. and Pinton P. (2014) Oncogenic and oncosuppressive signal transduction at mitochondria-associated endoplasmic reticulum membranes. Mol. Cell Oncol. 1, e956469 10.4161/23723548.2014.956469 27308328 PMC4905193

[39] Bruning A., Brem G.J., Vogel M. and Mylonas I. (2014) Tetracyclines cause cell stress-dependent ATF4 activation and mTOR inhibition. Exp. Cell Res. 320, 281–289 10.1016/j.yexcr.2013.11.012 24280420

