# Supplementary material for: Mitochondrial translation inhibition triggers ATF4 activation, leading to integrated stress response but not to mitochondrial unfolded protein response
Source: Biosci Rep. 2020 Nov 24;40(11):BSR20201289. doi: 10.1042/BSR20201289 (PMC7685009; doi:10.1042/BSR20201289)
Supplement: Supplementary Tables S1-S2 [file BSR-2020-1289_supp.pdf]

Supporting information Table 1

Primer sequence: The sequences of the qRT-PCR primers are as follows

Primer	Forward (5’ - 3’)	Reverse (5’ - 3’)
Gdf15	cttgaagacttgggctggag	taagaaccaccggggtgtag
Fgf21	gggaggatggaacagtggta	gtcctccagcagcagttctc
Cdsn	cctcctcgtcttttcctggt	tggttctcaggcgatggatt
Trib3	gctgtgggattcaagccaaa	ctgtgggcctgggtactaaa
Atf3	aactggcttcctgtgcactt	ggccagctaggtcatctgag
Slc7a5	cacctgccttctgtcctctc	tgaatcggagccacatcata
Sesn2	tagcctgcagcctcacctat	ctacgggtcgtcttctcagg
Angptl6	gcccactacgacagcttctc	gaggttagagtgggcacagg
Atf4	tcgatgctctgtttcgaatg	agaatgtaaagggggcaacc
Hsp60	aaagatggggtcactgttgc	catcacacctctccggattt
Hsp70	tgctgatccaggtgtacgag	cgttggtgatggtgatcttg
Hsp10	ggtcaggagggaaaggaaag	cagcttcacgtgacaccatt
ClpP	tgatcgagtcagcaatggag	cccagcagaggaagtttcag
Chop	cagaggtcacacgcacatcc	ccttgctcttcctcctcttcc
Gadd45a	atggcatccgaatggaaata	ttctcgcagcttccttcttc
18S	cgcggttctattttgttggt	agtcggcatcgtttatggtc
Acot2	attgggctgcttgggatttc	agcttccacgacatccaaga
Bcat2	gcatctagtccagcgtcctc	ccaaggttctcccttgaaca
Cebpb	cgcaacacacgtgtaactgtc	cgaaacggaaaaggttctca

Supporting information Table 2

Primer sequences of the ChIP primers are as follows

Primer	Forward (5’ - 3’)	Reverse (5’ - 3’)
Fgf21	TGCTCAGGGTTCCTCCTAGA	ACCAACCCCTGCTTAGCATT
Gadd34	GATGTTGGCGCAGATTGAGT	GGACGCAATGTTTCACAGG
Atf3	ATTACGTCAGCCTGGGATTG	CTCGCTGAGTGAGACTGTGG
Atf6	CTGGCTTTCCTCTCACCTTG	TAGCAAACCAAGTGCAGCAG
Trib3	GGGCGGGTCACAGATGGTGC	GACCGCCGCCAGCCTAACTG
Sestrin2	CTCCTTGGGCTGTCACTCTC	GTCCAATCAGCATCGACAAG
Cdsn	TTCTGGGTTAGGCAATGGAG	AGTTGGTGTGGCAGGAGAAG
Bcat2	GTAAGGGGTGGAACTGCAAA	GGAACGCACAACAGTAAACG
IL6 exon	TTCCATCCAGTTGCCTTCTTG	AGGTCTGTTGGGAGTGGTATC
